# Non-Coding RNAs Extended Omnigenic Module of Cancers

**DOI:** 10.3390/e26080640

**Published:** 2024-07-27

**Authors:** Jie Li, Bingbo Wang, Xiujuan Ma

**Affiliations:** School of Computer Science and Technology, Xidian University, Xi’an 710119, China; ljie@stu.xidian.edu.cn (J.L.); xjuanma@stu.xidian.edu.cn (X.M.)

**Keywords:** cancer module, connectivity pattern, heterogeneous network, non-coding RNAs

## Abstract

The emergence of cancers involves numerous coding and non-coding genes. Understanding the contribution of non-coding RNAs (ncRNAs) to the cancer neighborhood is crucial for interpreting the interaction between molecular markers of cancer. However, there is a lack of systematic studies on the involvement of ncRNAs in the cancer neighborhood. In this paper, we construct an interaction network which encompasses multiple genes. We focus on the fundamental topological indicator, namely connectivity, and evaluate its performance when applied to cancer-affected genes using statistical indices. Our findings reveal that ncRNAs significantly enhance the connectivity of affected genes and mediate the inclusion of more genes in the cancer module. To further explore the role of ncRNAs in the network, we propose a connectivity-based method which leverages the bridging function of ncRNAs across cancer-affected genes and reveals the non-coding RNAs extended omnigenic module (NeOModule). Topologically, this module promotes the formation of cancer patterns involving ncRNAs. Biologically, it is enriched with cancer pathways and treatment targets, providing valuable insights into disease relationships.

## 1. Introduction

The development of cancers involves multiple variant sites which affect numerous coding and non-coding genes. Most variants are located in non-coding regions and have small effect sizes on cancers [1,2]. Many ncRNAs play an important role in regulating the complex molecular and biological processes of cancer. Simultaneously, ncRNAs can be categorized into several types based on their size. The common types include long non-coding RNAs (lncRNAs), microRNAs (miRNAs), and pseudogenes. Existing studies indicate that miRNAs can regulate both coding genes and ncRNAs, including oncogenes such as RAS and WNT, as well as tumor suppressor genes such as TP53 and PTEN [3]. Moreover, this regulatory relationship can promote cancer development. For example, miR-31 targets genes such as RAS and WNT, thus accelerating cell proliferation and metastasis in lung cancer [4]. Extensive in vivo experiments have demonstrated that certain ncRNAs function as tumor suppressors or oncogenic factors. For example, lncRNA MEG3 can increase the expression level of p53 to activate apoptosis and reduce the proliferation of lung cancer [5]. Aside from this, recent studies have found that ncRNAs can function as biomarkers for cancer diagnosis [6]. Therefore, in-depth study of ncRNAs may offer new insights into cancer development and treatment.

The omnigenic model of genetic architecture suggests that cancer risk is influenced by the combined effects of core gene variants and peripheral gene variants. Core genes have a strong effect on the occurrence of cancer, while peripheral genes have a relatively weak effect value. Peripheral genes regulate core genes, which directly impact cancer development through a network. Additionally, the majority of variants with weak effects are found in non-coding regions [7]. The occurrence of cancer is often associated with mutations in multiple genes and interactions between genes [8]. The Cancer Genome Atlas (TCGA) [9] provides publicly available cancer genome datasets for research. Additionally, research on gene interactomes is becoming more mature, and there are many databases providing experimentally validated gene–gene interactions [10,11]. These interactions also reflect many physiological and pathological processes, such as cell proliferation and differentiation [10]. To study cancer neighborhoods, a general model is used to construct an interaction network, where a specific neighborhood represents a subgraph in the network [12]. On the one hand, the cancer neighborhood can elucidate the positioning of cancer-related genes in networks and unveil the relationship among cancers. For example, cancers with overlapping neighborhoods show significant symptom similarities and comorbidity characteristics [13]. Therefore, studying cancer neighborhoods based on interaction networks is crucial for understanding cancer mechanisms and treatment strategies.

However, current research on cancer neighborhoods primarily focuses on mesoscale subgraphs composed of some coding genes. There is no clear definition or description of neighborhoods involving ncRNAs. Some studies propose that coding genes affected by cancer form densely connected local subgraphs, known as cancer modules. Existing methods for identifying cancer modules using network models are mostly based on this assumption. For example, Zhou et al. [14] used a clustering approach to detect dense clusters in co-expression networks as cancer modules and predicted potential cancer prognostic genes. Some researchers have expanded the concept of cancer modules to include ncRNAs closely associated with oncogenes, thus incorporating ncRNAs into the neighborhood [15]. However, the hypothesis that cancer-related coding genes and ncRNAs are closely linked to form regional modules has not been fully substantiated. In fact, opposing findings argue that subgraphs of cancer-coding genes are fragmented [16]. The credibility of denseness as a characteristic of cancer genes in a network and the extent of ncRNA involvement in cancer neighborhoods remain to be further validated.

Current studies on ncRNAs in cancer mainly include, among other subjects, the association between ncRNAs and cancer and the functional annotation of ncRNAs [17,18]. These studies uncovered ncRNAs associated with cancer and provided new insights into the study of cancer-related biological processes. To the best of our knowledge, there have been limited systematic studies on the involvement of ncRNAs in cancer neighborhoods, despite their importance in understanding the impact of ncRNAs on cancer. It is widely recognized that cancer development is closely linked to genetic variations and abnormal gene expression. In addition, some studies utilize differential gene expression patterns between normal and cancer samples to investigate cancer-affected genes [19]. In addition, the expression level is a key factor in the development of ncRNA-based diagnosis and therapy. Previous studies also pointed out that abnormal expression of miRNAs can alter multiple apoptotic pathways, leading to the occurrence of cancers [20]. Therefore, analyzing gene expression data can help identify potential diagnostic markers and critical oncogenes, with significantly differentially expressed genes considered to be cancer-affected genes. Understanding how ncRNAs regulate these genes remains an important issue in the field of anti-cancer research.

This paper investigates the interaction pattern between cancer-affected non-coding genes and coding genes using an interaction network (IN). We explore the role of ncRNAs in the omnigenic cancer neighborhood based on topological metrics, including density, conductance, spatial network association, and connectivity. We observe significant differences in the connectivity metrics with and without the participation of ncRNAs in a cancer neighborhood. This suggests that the cancer neighborhood is a fragmented subgraph formed by cancer-affected coding genes. But in this subgraph, ncRNAs act as bridges between coding genes, revealing the topological regularity of ncRNAs in the cancer neighborhood. We introduce a connectivity-based method, labeled NeOModule, to identify the cancer neighborhood involving participation of ncRNAs. Additionally, through biological function enrichment analysis, we discover the role of ncRNAs in enhancing and facilitating the formation of pathways related to cancer. We also find that NeOModule can effectively describe cancer relationships in practical applications.

## 2. Materials and Methods

**Figure 1:**
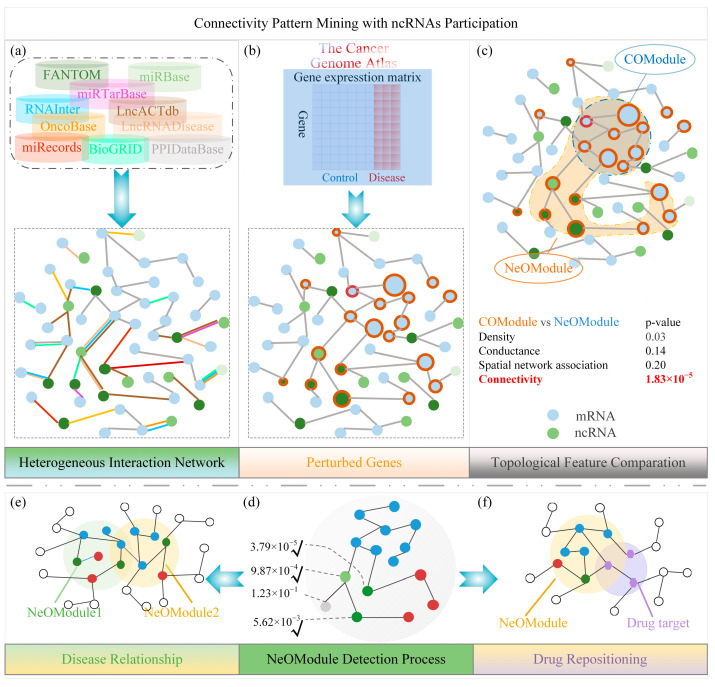
Schematic diagram of cancer neighborhood with participation of ncRNAs. (**a**) Construction of an interaction network (IN) using data from multiple sources. The blue and green circles represent coding genes (mRNAs) and ncRNAs, respectively. A multicolored edge indicates its existing in multiple databases. (**b**) Perturbation degree calculated by fold change of genes in a cancer state based on expression data from The Cancer Genome Atlas (TCGA). We circled the considerably affected genes under some sophisticated given thresholds, and the sizes of the nodes are proportional to the fold change values. (**c**) Comparation of the topological features of the Coding Omnigenic Module (COModule) and NeOModule. (**d**) A connectivity-based method to detect cancer neighborhoods with ncRNA participation. Red nodes represent Iso_mRNAs. (**e**,**f**) Applications of the NeOModule in cancer relationship analysis and drug repositioning. Purple nodes indicate drug targets.

To investigate the interaction between cancer-affected genes in the entire genome and the resulting neighborhood, we followed five steps. (1) A heterogeneous interaction network (IN) including multiple gene types (see Supplementary Note S1 in Supplementary Material) was constructed. (2) We performed differential expression analysis of the gene expression values between cancerous and normal samples. Subsequently, we projected the differentially expressed genes onto the IN to obtain the subgraphs which were affected by cancer. (3) We quantified the topological features of these subgraphs within the IN. (4) We identified the cancer neighborhood involving ncRNAs. (5) Finally, we explored the roles of ncRNAs within the cancer neighborhood (Figure 1).

First, we obtained the interactions from various sources, including FANTOM [21], miRBase [22], human interactome [23], OncoBase [24], LncACTdb [25], RNAInter [10], LncRNADisease [26], miRecords [27] and miRTarBase [28]. Specifically, we used the union of genes in FANTOM, the miRNAs in miRbase, and the genes in PPI as background nodes in the network and added the union of interactions in the above multiple databases as edges in the network. Finally, we obtained a heterogeneous interaction network (IN) with 24,215 nodes and 314,748 edges (supplementary data S1.xlsx). In the IN, the interactions between coding genes accounted for about 87.39% of the total, the interactions between ncRNAs accounted for 11.49%, and the interactions between non-coding and coding genes accounted for 1.12%. Most of the nodes in IN have a small degree, and only a few nodes have a relatively large degree, which is consistent with the real biological network. Details of the interactions in the IN are shown in Figure S1.

Next, to study the cancer neighborhood involving ncRNAs across various cancers and systematically verify its universality, we collected expression data from The Cancer Genome Atlas (TCGA) for 12 cancers (details in Supplementary Note S2, Data 2.xlsx) with high incidence and mortality rates [29]. We performed differential expression analysis of the genes using the DESeq2 [30] and Limma tools [31] for the expression data and quantified the perturbation degree of gene *g* (fold change $f_{g}$; see Appendix A.2) in a cancer state. Each cancer had an average of 3344 considerably affected genes under sophisticated given thresholds, including nearly 2995 differentially expressed coding mRNAs (DE_mRNAs) and 349 differentially expressed non-coding RNAs (DE_ncRNAs, 106 lncRNAs, 195 miRNAs, and 48 pseudogenes). Then, from the omnigenic perspective, we analyzed the topological structural properties of the subgraphs composed of many cancer-related genes and the interactions among them. We tested the results under an optional perturbation threshold β for the coding genes. In order to facilitate the subsequent description, we will use the following terms and abbreviations.

DE_mRNA is the affected coding gene set in a cancer state, in which $\mathrm{DE}\text{\_}\mathrm{mRNAs}\left(\beta \right)=\left\{g|g\in \mathrm{mRNAs},f_{g}\geq \beta \right\}$, and this comprises all coding genes in the IN with a perturbation degree $f_{g}\geq \beta$.

DE_ncRNA is the affected non-coding gene set in a cancer state, in which $\mathrm{DE}\text{\_}\mathrm{ncRNAs}=\left\{g|g\in \mathrm{ncRNAs},f_{g}\geq \gamma \right\}$, γ are given sophisticated thresholds for lncRNAs, miRNAs and pseudogenes respectively.

COModule stands for the Coding Omnigenic Module, which is the induced largest connected component (LCC) of DE_mRNAs in the IN network. The LCC is an interconnected functional subgraph structure formed by cancer perturbation nodes and is a commonly used representation in disease module studies [32]. Here, we have COModule(β)≜LCC of DE_mRNAs(β).

NeOModule stands for the Non-Coding RNAs Extended Omnigenic Module, which is the LCC of DE_mRNAs(β) ∪DE_ncRNAs in the IN, indicating the neighborhood after importing DE_ncRNAs. where NeOModule(β)≜LCC of DE_mRNAs(β) ∪DE_ncRNAs.

Iso_mRNA represents the isolated coding gene set, in which genes do not belong to the COModule but are connected and extended by ncRNAs into the NeOModule.

sLCC represents our quantifying the connectivity of a subgraph by measuring the size of the largest connected component (sLCC).

Our main focus was to monitor the alterations in the topological features of the COModule derived from DE_mRNAs in the IN alone, with the NeOModule formed by adding DE_ncRNAs. Across the 12 cancers, we noted that 70.28% of the DE_mRNAs were interconnected in the IN, forming the COModule. After incorporating ncRNAs, 75.64% of the genes involved with each other constituted a significantly larger subgraph, namely the NeOModule.

We used several topological indicators such as density, conductance and spatial network association [16] (spatialNA) to measure the subgraphs (see Appendix A.3). Density quantifies the denseness of the internal edges in the subgraph, while conductance represents the degree of interaction between the internal nodes and external nodes in the subgraph (see Supplementary Note S3 in Supplementary Material). We compared the results with 1000 random subgraphs as counterparts and calculated the significance using the Z-score (see Appendix A.1). Research has shown that the topological characteristics of random graphs in disease modules exhibit normal distribution characteristics, such as the LCC size [33,34]. Based on the assumption of a standard normal distribution, we believe that if the statistical significance of a certain topological value has a Z-score≥1.65 (one-sided test empirical *p*-value < 0.05), then this means that the subgraph performs significantly better than the random counterparts in terms of that specific topological feature.

**Figure 2:**
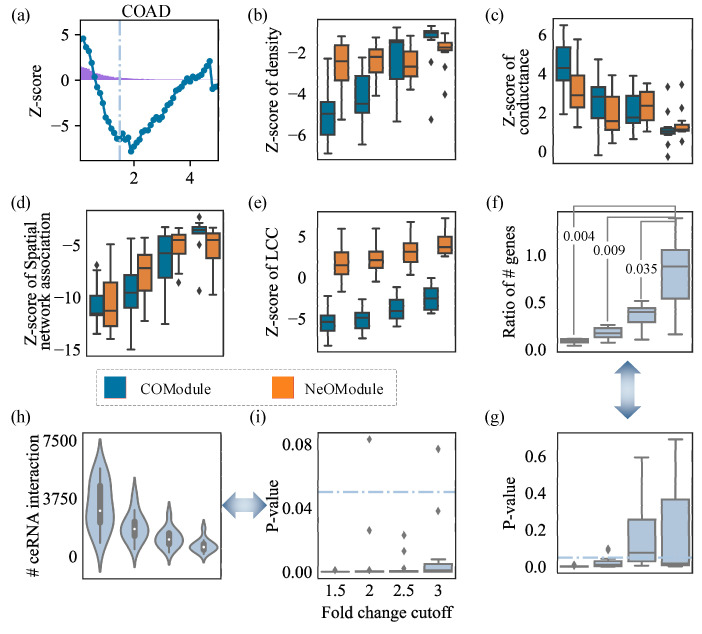
Topological characteristics of cancer omnigenic neighborhood. All abscissa values represent the perturbation cutoffs β. (**b**–**g**) Four groups of results for β=1.5,2,2.5,3. (**a**) Curve of the connectivity significance sLCC Z-scores of DE_mRNAs(β) in COAD. The light blue line corresponds to the most fragmented position at about β=1.5. The purple bars show the frequency distribution of the affected degree values (|logFC|≠0). (**b**–**d**) The statistics of density, conductance, and spatialNA for the COModules and NeOModules. (**e**) The connectivity significance sLCC Z-scores for the COModules and NeOModules. (**f**) The ratio of Iso_mRNAs in NeOModules, with the *p*-values comparing results between β=1.5 and β∈{1.5,2,2.5}. (**g**) Significance of the number of Iso_mRNAs at each perturbation threshold. (**h**) Number of triples (ncRNAs-miRNAs-mRNAs) in NeOModules. (**i**) Statistical significance *p*-value for the number of triples in the NeOModules.

As shown in the schematic diagram in Figure 2a, for COAD, 4894 DE_mRNAs under a low perturbation threshold β=1 formed COModule(1) (sLCC = 4090, Z-score = −4.10). When increasing the perturbation threshold, 2918 DE_mRNAs under a medium perturbation threshold β=1.5 formed COModule(1.5) (sLCC = 2014, Z-score = −6.55). Then, 156 DE_mRNAs formed COModule(4.7) under a high perturbation threshold of β=4.7 (sLCC = 16, Z-score = 2.08). The Z-score was lower than zero, indicating that the DE_mRNAs were relatively more fragmented and did not exhibit significant connectivity characteristics. In the 12 cancers we studied, we observed that when the threshold β was approximately 1.5, the connectivity significance of the omnigenic DE_mRNAs reached its floor values and showed obvious fragmentation (Figure S2). The connectivity significance reached its peak when β was about 4, and the Z-score was higher than that of the random gene sets, indicating noticeable connectivity. However, there were only 16 DE_mRNAs which were most correlated with COAD risk in a connected component among the 156 highly affected genes. Next, we examined the performance of the average statistics of density, conductance, and spatialNA. For the COModules of 12 cancers (Figure 2b–d), the average ${\text{Z-score}}_{\mathrm{density}}=-5.05$, ${\text{Z-score}}_{\mathrm{conductance}}=4.51$, and ${\text{Z-score}}_{\mathrm{spatialNA}}=-10.71$. This means that the DE_mRNAs showed loose connections within the COModule, frequently interacting with outside genes, and the degree of aggregation was significantly low as β∈{1.5,2,2.5,3}. These findings challenge the conclusions of studies which relied on the denseness hypothesis but confirm that the COModule did not have the characteristics of being tightly connected within and loosely connected outside the module. Another question we addressed was whether these topological features would remain the same after adding ncRNAs to the omnigenic module or not. Then, we explored the role of ncRNAs in a cancer omnigenic neighborhood. We measured the topological metrics of the NeOModule. The average statistics for density, conductance, and spatialNA in the 12 cancers were ${\text{Z-score}}_{\mathrm{density}}=-2.77$, ${\text{Z-score}}_{\mathrm{conductance}}=3.15$, and ${\text{Z-score}}_{\mathrm{spatialNA}}=-10.62$, respectively (Figure 2b–d). However, the connectivity of the NeOModules exhibited significant divergence from the COModules (Figure 2e and Figure S3). Specifically, the Z-score of the sLCC for NeOModule(1.5) improved by 7.15 compared with the significant fragmentation in COModule(1.5) on average ($p\text{-value}=1.83\times 10^{-5}$). The Z-score of the connectivity’s sLCC for NeOModule(3) improved by an average of 6.86 ($p\text{-value}=1.83\times 10^{-5}$) compared with that of COModule(3). The ncRNAs synergized with the COModules and connected the Iso_mRNAs. In other words, ncRNAs play the role of improving connectivity and making more coding genes participate in NeOModules. This connectivity pattern is the underlying property of ncRNAs in cancer neighborhoods rather than other density-based ones.

Furthermore, we calculated the proportion of Iso_mRNAs introduced by ncRNAs. The results indicate that in over half of the 12 cancers, more imported mRNAs were introduced in the NeOModules (Figure 2f,g). For the high perturbation thresholds in particular (β=3), the NeOModule was extended significantly more than for the low perturbation thresholds. This indicates that ncRNAs play a substantial role in expanding the highly affected regions of DE_mRNAs. In our tests, the number of lncRNAs participating in the NeOModules ranged from 56 to 92, the number of miRNAs ranged from 59 to 310, the number of pseudogenes ranged from 12 to 37, and the number of imported Iso_mRNAs ranged from 139 to 216. These ncRNAs are genes which can link DE_mRNAs and Iso_mRNAs to form a connected disease module. Additionally, many triple competing endogenous RNA (ceRNA) interactions which have been studied are thought to be closely related to gene regulation and disease [25,35]. We observed that significantly more triples (ncRNAs-miRNAs-mRNAs) were formed in the NeOModules (β=3, rank-sum test $p\text{-value}=8.33\times 10^{-5}$), indicating that ncRNAs as connector were more likely to participate in the functional triples, which in turn formed detectable ceRNA interactions.

Based on these observations and analysis, we proposed a connectivity-based method (see Appendix A.5) to mine the cancer omnigenic neighborhood with ncRNA participation.

## 3. Results

### 3.1. ncRNAs Expand Cancer Pathways

**Figure 3:**
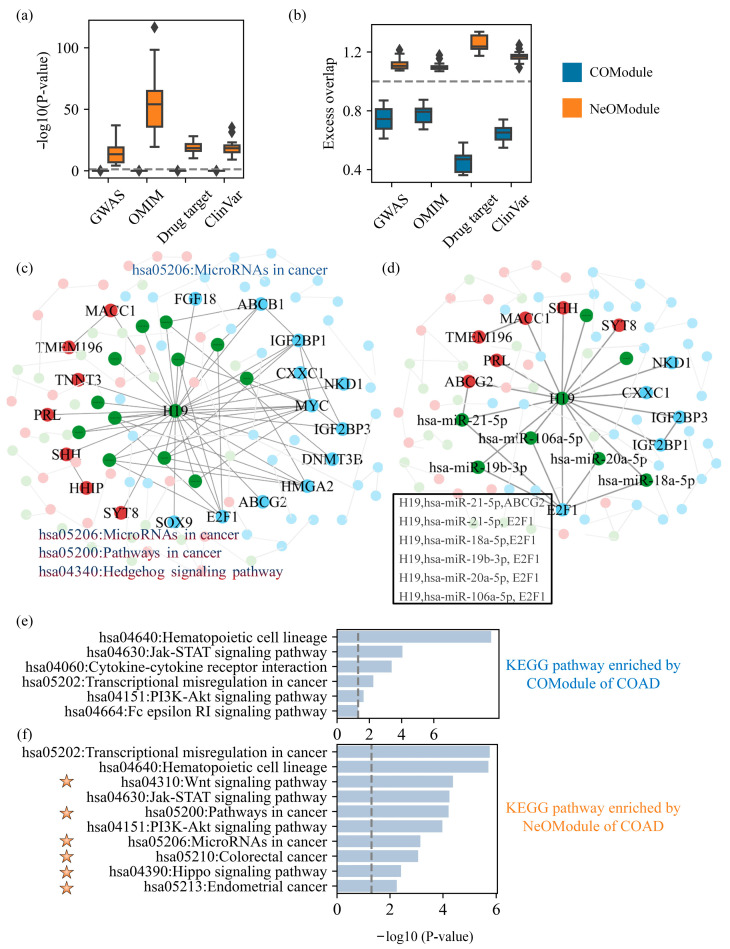
The function of the NeOModule in cancer and the role of ncRNAs. (**a**,**b**) The enrichment of the NeOModule and COModule in different functional gene sets. (**c**,**d**), The H19-centered subgraphs in COAD, which are ${\mathrm{NeOModule}}_{H19}\left(1.5\right)$ and ${\mathrm{NeOModule}}_{H19}\left(2\right)$. Blue, green, and red nodes represent DE_mRNAs, DE_ncRNAs, and Iso_mRNAs, respectively. (**e**) KEGG pathways significantly enriched by the COModule of COAD. (**f**) KEGG pathways significantly enriched by the NeOModule of COAD. KEGG pathways with an orange pentagram on the left were more significantly enriched by the NeOModule than the COModule.

To explore the function of the NeOModule in cancers, we conducted enrichment analysis of the genes in the NeOModule for each cancer by utilizing four established functional gene datasets (Table 1). The results (Figure 3a,b) revealed significant enrichment of the disease-related genes in the NeOModule, underscoring its importance in elucidating disease development and potentially offering insights into cancer relationships. Additionally, the genes within the NeOModule showed significant enrichment of cancer drugs, suggesting that the NeOModule was relevant to cancer therapy and might help us carry out drug repositioning with known drugs. Then, we conducted KEGG pathway analysis on the NeOModules of cancers and found that they could be enriched with significant cancer-related functional pathways. For example, we found that the NeOModule of BRCA was significantly enriched in the PI3K-Akt signaling pathway, with a $p\text{-value}=6.17\times 10^{-5}$. This is an oncogenic signaling pathway of widespread concern [36]. Interestingly, it was also significantly enriched in the systemic lupus erythematosus (SLE) pathway, with a $p\text{-value}=5.20\times 10^{-15}$, suggesting that patients with BRCA are also at risk of SLE. Previous studies have also suggested that SLE may be associated with BRCA and pointed out that patients with SLE may have reduced risk of BRCA [37]. Therefore, the genes in the NeOModule were not only related to cancer but also considerably enriched in some pathways associated with cancer. However, another critical question is which effect ncRNAs have on the function of the NeOModule.

To investigate the function of ncRNAs in the NeOModule and their association with cancer, we conducted pathway enrichment analysis on the genes in a specific NeOModule. Specifically, by comparing the differences in the pathways enriched by gene sets without and with the participation of ncRNAs, we analyzed whether new pathways emerged and if the originally enriched pathways changed. Also, we investigated the reasons underlying these pathway changes. Initially, we extracted the NeOModule of COAD when β=1.5, focusing on H19, one of the lncRNAs studied the earliest [38], to generate a subgraph represented by ${\mathrm{NeOModule}}_{H19}(1.5)$ (Figure 3c). We confirmed that the overexpression of H19 not only posed a risk factor for reducing the survival in patients with colon cancer but was also associated with the cell proliferation and metastasis of colon cancer cells. Next, we used DAVID [39] to conduct KEGG pathway enrichment analysis for the genes in ${\mathrm{COModule}}_{H19}(1.5)$ and expanded the coding genes in ${\mathrm{NeOModule}}_{H19}(1.5)$. We regarded pathways with a p-value≤0.05 as significantly enriched pathways. We found that some coding genes in ${\mathrm{COModule}}_{H19}(1.5)$ were only enriched in microRNAs in the cancer pathway hsa05206. As a result of H19’s involvement, several Iso_mRNAs were integrated into the subgraph, leading to the enrichment of coding genes in ${\mathrm{NeOModule}}_{H19}(1.5)$ not just in hsa05206 but also in two novel pathways, namely the hsa05200 cancer pathway and the hedgehog signaling pathway hsa04340. The hsa05200 pathway contained two Iso_mRNAs, which were SHH and HHIP. This led to ${\mathrm{NeOModule}}_{H19}(1.5)$ being enriched in hsa05200. Furthermore, previous studies have implicated hsa04340 in colon cancer [40,41]. With regard to SHH and HHIP, Gerling et al. [40] pointed out that SHH is up-regulated in colon cancer (logFC(SHH)=2.191), and its expression correlates with the treatment of COAD. HHIP (logFC(SHH)=−1.751) was also confirmed to have reduced expression in COAD patients [41]. In short, the participation of ncRNAs enriches cancer pathways beyond consideration of the coding genes in DE_mRNAs alone. This facilitates the identification of cancer-related pathways.

Next, we obtained another H19-centered subgraph with 19 genes, which was ${\mathrm{NeOModule}}_{H19}(2)$, in COAD (Figure 3d). We analyzed all triples involved in the subgraph one by one. The structure of the triples here was in the form of lncRNA-miRNA-mRNA. A total of six triples were involved in this subgraph, including one lncRNA, five miRNAs, and two mRNAs. These five miRNAs all showed significantly low expression (logFC≤−1), and all of them were verified to be associated with colon cancer in the dbDEMC [42] and MNDR [43] databases. Among them, miR-18a, miR-19b, and miR-20a belong to the miR-17-92a cluster, which is usually described as an oncogene [44]. We also mentioned that H19 was highly expressed in COAD. According to the hypothesis of ceRNAs [45], when the miRNA in a triple is expressed less, and one RNA which interacts with the miRNA is highly expressed, the mRNA which interacts with the miRNA should be highly expressed. Here, the two mRNAs were ABCG2 and E2F1. E2F1 (logFC(E2F1)=1.757) was indeed highly expressed in COAD, and it has been shown to be involved in the proliferation and apoptosis of colon cancer cells [46]. Therefore, we inferred that H19 and E2F1 can compete to bind these five miRNAs, and such a ceRNA relationship may be associated with COAD. Another mRNA ABCG2 (logFC(ABCG2)=−5.075) was expressed less in colon cancer. Although it was pointed out that ABCG2 is related to colon cancer, the differential expression of this gene plays an important role in the photodynamic therapy of colon cancer [47], and studies have shown that there is still controversy over the expression of ABCG2 [48]. Therefore, the NeOModule can help us explain the incidence and diagnosis of cancer using the ceRNAs associations formed by cancer-related factors.

To delve deeper into the function of ncRNAs and underscore their importance in cancer, we curated cancer modules from previous studies and extended the collected modules through the IN and DE_ncRNAs related to cancer. Furthermore, we compared the pathways enriched by the COModules and NeOModules. We first obtained a COAD module containing six coding genes [49]. The modules before and after expansion were recorded as the COModule of COAD and the NeOModule of COAD, respectively. Then, KEGG enrichment analysis was performed on both modules. Lastly, we found the top 10 significantly enriched pathways (Figure 3e,f). We found that compared with the pathways enriched by the COModule, the NeOModule of COAD had three significantly enhanced pathways, namely hsa04630, hsa04151, and hsa05202, which have been confirmed to be related to cancers [34,49,50]. Among them, hsa05202 is a transcriptional dysregulation pathway in cancer, and it has been considered to be the main cause of abnormal phenotypes in tumor cells. Additionally, hsa05202 was thought to effectively distinguish cancer-related and unrelated lncRNAs [51]. New pathways enriched by the NeOModule (pathways not significantly enriched by the COModule) included hsa04310, hsa05200, hsa05206, hsa05210, has04390, and hsa05213, of which the first five pathways were all considered to be related to colon cancer [34,52,53]. While the other pathway, hsa05213, relates to endometrial cancer, previous studies have shown that patients with endometrial cancer may have colon cancer at the same time [54]. For another pathway, hsa04640, which was weakened but still significant, we did not find an association between this pathway and cancer, and the relationship remains to be verified.

Additionally, we collected a BRCA module containing 35 coding genes [55], denoting the collected module as the COModule and the module expanded with ncRNAs as the NeOModule of BRCA. We analyzed several new pathways—hsa04110, hsa05206, and hsa04060—enriched by the NeOModule due to the participation of ncRNAs (Figure S4). For hsa04110, the ncRNAs in the NeOModule introduced mRNAs such as MCM2, which made it significantly enriched in this pathway. Previous studies have shown that cell cycle-based regulatory markers such as MCM2 and PHH3 can help identify tumors with poor prognoses but which respond well to systemic therapy [56]. For hsa05206, genes such as MMP9 and CCNE1 in this pathway are introduced by miRNAs in the NeOModule. Moreover, miR-497 (logFC=1.451) in breast cancer cells regulates the growth of cancer cells by targeting CCNE1 [57]. Liu et al. [58] found that genes such as CXCL10 (CXCL10 in NEM_BRCA is introduced by ncRNAs) may be involved in breast cancer neoadjuvant chemotherapy through the hsa04060 pathway. Therefore, the involvement of ncRNAs makes cancer-related pathways more prominent and rank higher in all pathways. This underscores the importance of considering ncRNAs in advancing cancer pathway research.

### 3.2. Application of NeOModules

To explore the advantages of the NeOModule in characterizing cancer, we utilized NeOModules of 12 different cancers to analyze their relationships. We quantified similarities based on the NeOModules, COModules, and Iso_mRNAs of these cancers. Taking the NeOModule as an example, we calculated the Jaccard coefficient between genes in the NeOModules of two cancers (see Appendix A.4).

**Figure 4:**
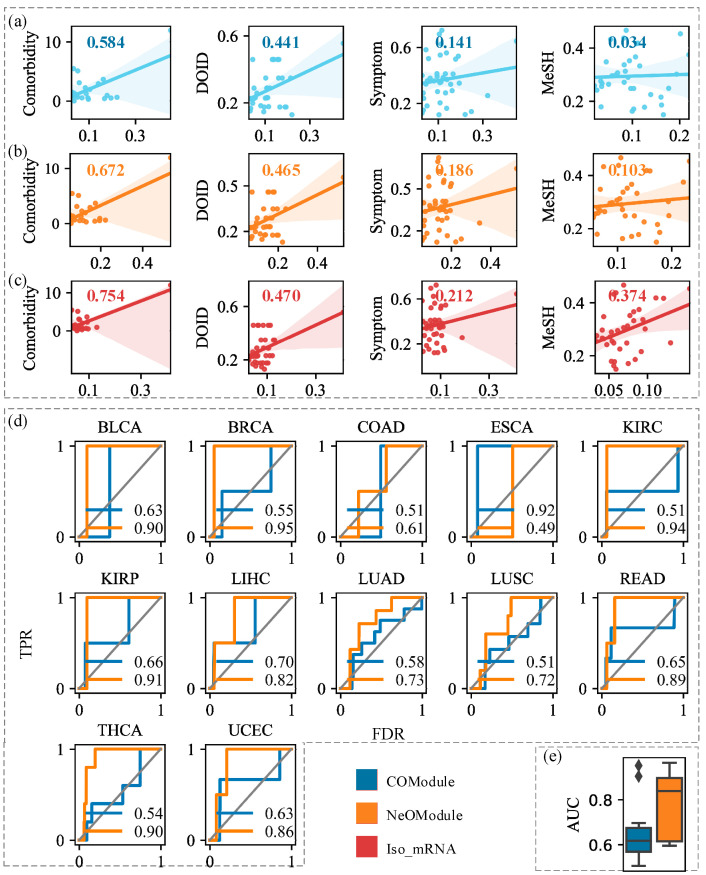
Application of NeOModule in understanding disease relationship. (**a**–**c**) Relationships between cancers characterized by COModules, NeOModules, and Iso_mRNAs of 12 cancers and the associations between similarities calculated by our methods and previous studies (β=2.5). The known similarity data used in the four columns from left to right are for comorbidity, DOID, symptoms, and MeSH similarity. The numbers in the upper left corner of each figure denote the Pearson correlation coefficients between the cancer-affected subgraphs and other established datasets. (**d**) ROC curves for the prediction of drugs to treat the corresponding cancers according to the COModules and NeOModules under different perturbation degrees. The numbers in the lower right corner are the corresponding AUC values. (**e**) AUC values obtained by COModules and NeOModules of 12 cancers in drug prediction when β=1.5.

In order to verify whether the cancer associations portrayed by the NeOModules were accurate, we obtained disease similarity data from four known sources (Table 2) as reference answers, including Medical Subject Headings (MeSH) [59], symptom similarity data [60], disease ontology similarity data [61], and disease comorbidity data [62]. Specifically, we conducted a correlation analysis between the known sophisticated similarities and our results (Figure 4a–c). The results show that compared with the COModules, the similarities between cancers obtained by the NeOModules and Iso_mRNAs showed greater relevance to existing studies. In particular, the correlation calculated by the comorbidity relationship was the highest. When β=2.5 (results under different perturbation thresholds in Figure S5), the correlations between the NeOModules and the four datasets mentioned above increased by 203.58%, 31.84%, 5.44%, and 15.06%, respectively, compared with the COModules. The Iso_mRNAss increased by 1001.95%, 49.90%, 6.53%, and 29.04%, respectively, compared with the COModules. Especially for the MeSH data, when we did not consider ncRNAs, the correlation ($r_{COModule}=0.03$) between cancers observed by the COModules and MeSH was rather weak, while the correlation observed by the NeOModules was greatly improved ($r_{\mathrm{NeOModule}}=0.10$, $r_{\mathrm{Iso}\text{\_}\mathrm{mRNAs}}=0.37$). This demonstrates that NeOModule can accurately depict the relationships between cancers, highlighting the critical role of considering ncRNAs in cancer research.

We considered whether drug prediction based on a NeOModule might reveal more accurate therapeutic relationships between drugs and cancer or not. We first collected FDA-approved drugs for 12 cancers from repoDB [63] and obtained a total of 107 cancer-drug association pairs between 12 cancers and 65 drugs. Drug target data were collected from a study by Cheng et al. [23]. Next, we calculated the distance through the NeOModules, COModules, and drugs. Then, we ranked the drugs according to the distance from small to large and verified whether the top-ranked drugs could be used to treat the corresponding cancers through 107 cancer-drug pairs. The results show that the NeOModule outperformed the COModule in terms of drug prediction (Figure 4d). In 12 cancers, the AUC of the NeOModules increased by 21.93%, 18.06%, 21.72%, and 22.57% compared with the COModules (results under different perturbation thresholds in Figure S6). Therefore, we believed that the distances between the cancers and drugs were changed because ncRNAs were involved in the cancer neighborhoods, thus improving the prediction accuracy of the drug-cancer treatment relationships. This further illustrates the importance of NeOModules in cancer research.

## 4. Discussion

We investigated a cancer neighborhood with the involvement of ncRNAs using an interaction network and cancer expression data. Initially, we constructed an IN comprising multiple types of genes. Secondly, several topological features were employed to characterize the properties of the COModule and the NeOModule under two different conditions. Then, we employed the Z-score to assess the effectiveness of topological features. It was found that only connectivity showed a significant difference between the two subgraphs. Based on connectivity, we defined the cancer neighborhood involving ncRNAs as a significantly connected and detectable subgraph formed by cancer-affected coding genes and ncRNAs. The ncRNAs played an important role in topologically connecting the fragment cancer-affected genes. Furthermore, we proposed a connectivity-based method to detect a cancer neighborhood NeOModule with ncRNAs for each cancer. The nodes in the NeOModules were significantly related to disease-related genes. Additionally, there were many important pathways contained in the NeOModules. The NeOModule showed a close relationship with cancer both at the node level and the pathway level. More importantly, ncRNAs enhanced the identification of cancer-related pathways at the biological level. We also found that the NeOModule was more effective in characterizing disease relationships than focusing only on coding genes.

Overall, this paper provides a new tool for cancer research. The results show that our method can effectively detect the NeOModule that characterizes cancer. However, there are still some potential problems which can be considered in follow-up works. First, we only considered differential expression genes. Currently, multiomics data of cancer are gradually being enriched, such as somatic mutation or methylation. These studies might bring us a further understanding of the relationship between ncRNAs and cancer. Second, only a small subset of ncRNAs was included in our study. The number of ncRNAs was about 23.65% of all genes in the IN, while previous studies have shown that only about 2% of the region in the human genome can encode proteins. On one hand, the naming of ncRNAs in separate databases is not strictly unified. On the other hand, the accumulation of experimentally verified ncRNA interaction information is relatively slow. Although there are many ncRNA-related interactions predicted by calculation tools, further verification is still needed. Last but not least, the selection of the threshold for perturbed cancer genes is still a topic worthy of discussion. However, the understanding of ncRNAs provides a new channel for us to further understand the mechanism of cancer and find drugs based on ncRNAs involved in cancer pathways. It is also quite necessary to consider ncRNAs in subsequent studies.

## Figures and Tables

**Table 1:** The information of the functional gene dataset.

Gene Set	Number of Genes	Source
GWAS	19,110	http://www.ebi.ac.uk/gwas/ (accessed on 10 September 2016)
OMIM	16,291	https://omim.org/ (accessed on 10 September 2016)
ClinVar	5420	https://www.ncbi.nlm.nih.gov/clinvar/ (accessed on 10 September 2016)
Drug Target	2256	Network-based prediction of drug combinations

**Table 2:** The number of genes in the disease similarity data.

Disease Similarity Data	Number of Genes
Symptom similarity	1596
Disease ontology similarity	1125
Comorbidity data	376
MeSH	5080

**Figure A1:**
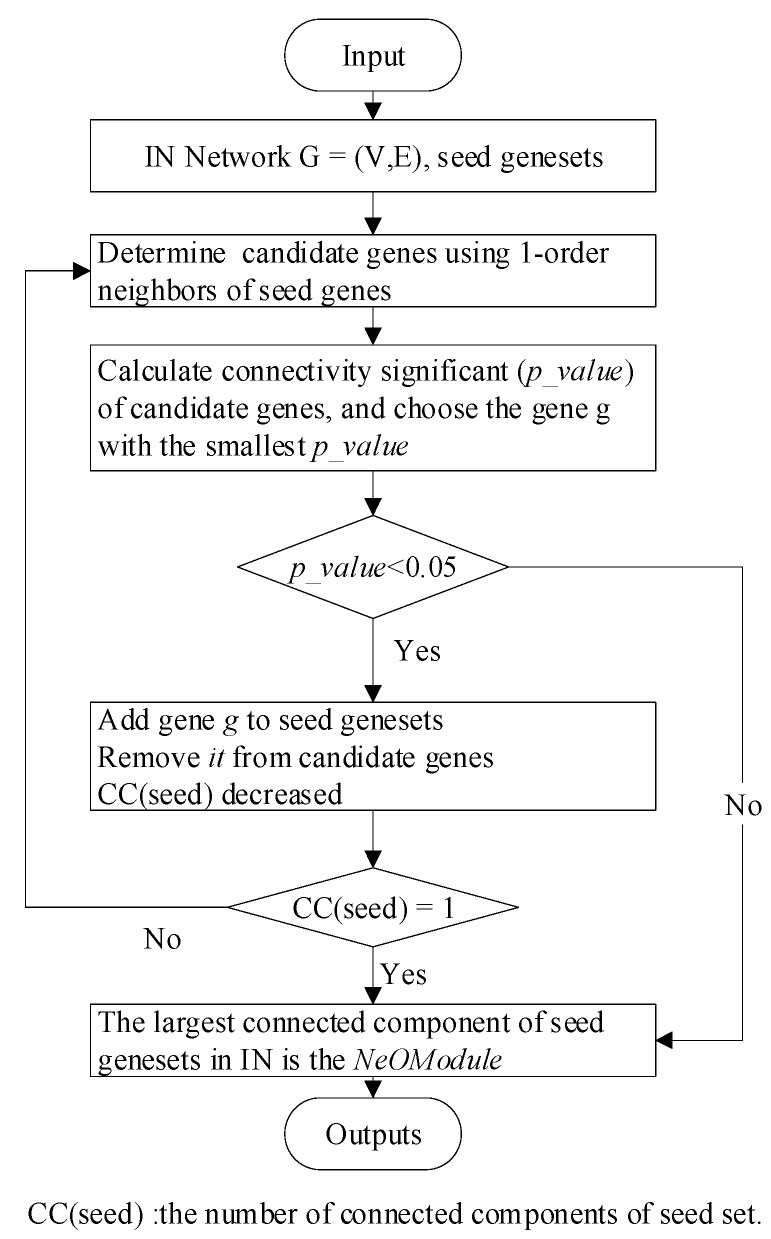
Flowchart of the NeOModule detection process.

## Data Availability

All supporting files can be downloaded from https://github.com/wangbingbo2019/NeOModule, including the heterogeneous interaction network (Supplementary Data S1.xlsx); differential expression for 12 cancers (Supplementary Data S2.xlsx); and NeOModule for 12 cancers (Supplementary Data S3).

## Supplementary Materials

The following supporting information can be downloaded at https://www.mdpi.com/article/10.3390/e26080640/s1. Supplementary Note S1: Interaction Network; Supplementary Note S2: Cancer-Related Expression Data; Supplementary Note S3: Analysis of Topological Features; Supplementary Note S4: Functional Profile of NeOModule; Supplementary Note S5: Subgraph Centered on H19 in NeOModule; Supplementary Note S6: Module Expansion; Supplementary Note S7: Distance between Cancer and Drug; Table S1: The number of samples involved in gene expression data; Table S2: The sources and numbers of the functional gene datasets; Figure S1: The details of interactions in the IN.; Figure S2: The connectivity curves of coding genes affected by each cancer; Figure S3: The topological features of COModule(β) and NeOModule(β); Figure S4: The top 10 pathways with the lowest *p*-values enriched by the NeOModule of BRCA; Figure S5: Associations between cancers characterized by the NeOModule, COModule and Iso_mRNAs of 12 cancers; Figure S6: Performance of the COModule and NeOModule in drug prediction for 12 cancers under different perturbation thresholds. References [64,65] are cited in Supplementary Materials.

## Appendix A

### Appendix A.1. Connectivity Significance Test

We use the size of the largest connected component (sLCC) to describe the connectivity of a subgraph. To measure whether the connectivity of a subgraph was significant, we compared the sLCC of the target subgraph with random subgraph. Finally, the connectivity significance of the target subgraph was quantified by the Z-score as follows:

(A1) $$\text{Z-score}=\frac{sLCC_{\mathrm{target}}-\mu (sLCC_{\mathrm{random}})}{\sigma (sLCC_{\mathrm{random}})},$$

where $sLCC_{\mathrm{target}}$ is the size of the largest connected component of the target subgraph, $sLCC_{\mathrm{random}}$ is the sLCC of the target subgraph, *μ* represents the mean of the sLCC of multiple random subgraphs, and *σ* is the variance. The method of selecting a random subgraph was as follows. For the target subgraph consisting of *k* nodes, we randomly selected *k* nodes in the IN to form a random subgraph. For the target subgraph composed of *m* mRNAs, we performed 1000 random selections in the IN, selecting *m* mRNAs each time to form a random subgraph, and we calculated the connectivity significance Z-score of the target subgraph based on the 1000 random subgraphs; For the target subgraph containing *m* + *n* nodes expanded by *n* ncRNAs, we randomly selected *m* + *n* nodes in the IN 1000 times, each time forming a random subgraph. A larger Z-score indicates that the cancer-related subgraph has more significant connected components compared with random subgraphs of the same node size in the network.

### Appendix A.2. Acquisition of Affected Genes

We computed the fold change index ($f_{g}=\left|\log_{2}FC\right|$) to quantify the differential expression levels, which represent the perturbation degree $f_{g}$ of a gene g in a state of cancer. According to the curve of the Z-score changing with $f_{g}$ for the coding gene set in various cancers (Figure 2a), we found that when $f_{g}=1.5$, the Z-score reached its minimum value. At this time, the cancer module reached maximum fragmentation. When $f_{g}>1.5$, the module gradually tended to be integrated, and thus we chose $f_{g}=1.5$ as the initial threshold in this study and continued to make the conditions more stringent, screening the set of coding genes that are significantly disturbed by cancer and expressing them as DE_mRNAs. Previous studies have shown that lncRNA expression is approximately 10 fold lower than that of coding genes [66]. Therefore, when obtaining the DE_mRNAs, we selected an lncRNA threshold of 0.15. Referring to a study by Xue Liu et al. [67], we set the selection threshold of the miRNAs and pseudogenes to 1.

### Appendix A.3. Topology Indicators

The density of a subgraph $G_{S}$ represents the denseness of the edges in it, which is usually calculated as follows:

(A2) $$\mathrm{Density}=\frac{2\left|E_{S}\right|}{\left|V_{S}\right|\left(\left|V_{S}\right|-1\right)},$$

where $E_{S}$ represents the number of edges in a subgraph $G_{S}$, and $V_{S}$ represents the number of nodes.

The conductance of a subgraph $G_{S}$ is used to measure the interaction degree between the internal nodes and external nodes, which is calculated as follows:

(A3) $$\mathrm{Conductance}=\frac{\left|B_{S}\right|}{\left|B_{S}\right|+2\left|E_{S}\right|},$$

where $B_{S}=\left\{\left(u,v\right)|\left(u,v\right)\in E,u\in V_{S},v\in V\backslash V_{S}\right\}$ is the boundary set of $G_{S}$ and V indicates the nodes in the IN. The smaller the conductance value is, the less the subgraph is connected to the outside.

A spatial network association of $G_{S}$ was used to measure the denseness of the nodes, which was calculated using the shortest distance between the nodes [1]:

(A4) $$\mathrm{SpatialNA}(l)=\frac{2}{{\left(|V_{S}|\right)}^{2}}\sum_{i}p_{i}\sum_{j}(p_{j}-\bar{p})I({ℒ}_{G}(i,j)<l)$$

If node i is in $G_{S}$, then $p_{i}=1$; otherwise, $p_{i}=0$. Meanwhile, $\bar{p}=|V_{S}|/n$, and n is the number of nodes in the IN. If the shortest path length between i and j is less than l, then $I({ℒ}_{G}(i,j)<l)=1$; otherwise, $I({ℒ}_{G}(i,j)<l)=0$. Here, a curve is formed by l from 2 to $l_{\max}$ and its corresponding value SpatialNA(l). The area under the curve is denoted by K(l). The larger K(l) is, the more the subgraph $G_{S}$ is aggregated in the IN. We set $l_{\max}=5$ in our experiments according to the distance of the nodes in the IN.

### Appendix A.4. Computing Similarity Metrics for Cancer Relationships

We quantified the similarity of the disease modules, such as the NeOModules, COModules, and Iso_mRNAs, to characterize the cancer relationships. Taking the NeOModule as an example, we calculated the Jaccard coefficient between nodes in the NeOModules of two cancers:

(A5) $${\mathrm{Jaccard}(\mathrm{NeOModule}}_{1}{,\mathrm{NeOModule}}_{2})=\frac{\left|{\mathrm{NeOModule}}_{1}\cap {\mathrm{NeOModule}}_{2}\right|}{\left|{\mathrm{NeOModule}}_{1}\cup {\mathrm{NeOModule}}_{2}\right|},$$

where ${\mathrm{NeOModule}}_{1}$ and ${\mathrm{NeOModule}}_{2}$ represent the node sets of the NeOModules corresponding to the two cancers, ${|\mathrm{NeOModule}}_{1}\cap {\mathrm{NeOModule}}_{2}|$ represents the number of elements in the intersection of the sets ${\mathrm{NeOModule}}_{1}$ and ${\mathrm{NeOModule}}_{2}$, and ${|\mathrm{NeOModule}}_{1}\cup {\mathrm{NeOModule}}_{2}|$ represents the number of elements in the union of sets ${\mathrm{NeOModule}}_{1}$ and ${\mathrm{NeOModule}}_{2}$. The closer the Jaccard coefficient is to 1, the more overlap the NeOModules of the two cancers have, and the more similar the cancers are.

In order to verify whether the cancer associations described by the NeOModule were accurate, we collected the existing cancer relationships of the 12 cancers in the study from four databases as the ground truth. We used $x_{1}=\{x_{11},x_{12},\ldots x_{1n}\}$ to represent the cancer associations described by our method, $x_{2}=\{x_{21},x_{22},\ldots x_{2n}\}$ to represent the cancer associations described in the known database, and n as the number of cancer relationship pairs. We calculated the correlation between vector $x_{1}$ and vector $x_{2}$ to characterize the similarity between our results and the known comorbidity. The closer the correlation was to 1, the more consistent the cancer associations described by our method were with the known associations.

### Appendix A.5. Detection of Cancer Neighborhood

Building upon the connectivity role of ncRNAs, we present a connectivity-based method (Figure A1) for detecting cancer neighborhoods involving ncRNAs. For a cancer type, we first obtained the DE_mRNAs. Then, we pinpointed the ncRNAs which serve a significant bridging function. Finally, we obtained the cancer neighborhood of the cancer. When the perturbation threshold of the genes was β for a certain cancer, the omnigenic and coding subgraphs were NeOModule(β) and COModule(β), respectively. For the NeOModule(β) of a cancer, we found the set of coding genes (seed set) and non-coding genes (candidate set). The connectivity significant to each node and edge in the network can be calculated using the C3 algorithm [68], which represents the ability of nodes or edges to significantly connect fragments in a network.
