# Supplementary material for: Non-Coding RNAs Extended Omnigenic Module of Cancers
Source: Entropy (Basel). 2024 Jul 27;26(8):640. doi: 10.3390/e26080640 (PMC11353529; doi:10.3390/e26080640)
Supplement: Supplementary file 1 [file entropy-26-00640-s001.zip › entropy-3023628-supplementary.pdf]

Supplementary Material

Supplementary Notes

Supplementary Note S1. Interaction Network

We collect and integrate data from the following multiple sources to construct the heterogeneous interaction network.

(1) RNAInter [1] (http://www.rna-society.org/rnainter/) database integrates experimentally validated and computationally predicted RNA-related interaction entries collected from the literature and other databases. This database includes lncRNAs, miRNAs and other types of RNA. We collect interactions supported by strong experimental evidence from RNAInter (download in 2020.8). Then, we preserve only the entries related to Homo sapiens. Finally, 26568 entries without repetition are obtained, where includes 10530 nodes.

(2) miRecords [2] (http://c1.accurascience.com/miRecords/) database integrates experimentally validated data collected from literatures, as well as predicted interactions between animal-related miRNAs and their targets. The targets are genes that can encode proteins. We collect experimentally verified interactions and obtain Homo sapiens-related entries (download in 2020.6, fourth edition). In order to avoid affecting results, we delete the co-expression interactions between miRNAs and targets. Finally, we get 1356 nodes and 1692 entries without duplicates.

(3) LncRNADisease [3] (http://www.cuilab.cn/lncRNAdisease) database integrates experimentally validated data collected from literatures, and the association between lncRNAs and human diseases predicted by computational tools. We collect experimentally verified interactions between RNAs (download in 2020.4, version 2015). In order to avoid the bias of follow-up experiments, we remove co-expressed interactions and end up with 148 entries without duplication, which contains 177 nodes.

(4) miRTarBase [4] (http://miRTarBase.cuhk.edu.cn/) database first performs natural language processing on the text to obtain articles related to miRNAs function, and then integrates interactions between miRNAs and targets obtained by literature mining. This database contains interactions for multiple species. We collect human-related entries and which are supported by strong experimental evidence in miRTarBase (download in 2020.6, version 8.0). Finally, we get 8489 entries without duplicates, including 3589 nodes.

(5) The BIOGRID [5] (https://thebiogrid.org/) database integrates physical and genetic interactions of genes/proteins, covering multiple species. We collect all interactions in BIOGRID (download in 2020.6, version 3.5.186), retaining only human-related genetic interactions. Finally, we get 3303 nodes and 8335 entries without duplication.

(6) The OncoBase [6,7] database integrates tissue-specific mutations related to somatic non-coding genes and 3D genomic data. We collect 3D genomic data, which includes three types of interaction data: promoter-promoter, promoter-enhancer, and enhancer-enhancer (download in 2018.8). We retain the promoter-enhancer interaction and finally get 65578 entries, including 22810 nodes.

(7) LncACTdb [8] (http://www.bio-bigdata.net/LncACTdb/index.html) database

integrates experimentally validated ceRNA interactions, including various RNAs such as lncRNAs and circRNAs. We collect entries related to human interaction (download in 2020.10), resulting in 2681 entries with 1668 nodes.

(8) Human interactome. There are 234714 protein-protein interactions and 16348 nodes collected from the study of Cheng et al. [9]. Since proteins are encoded by genes and there is a corresponding relationship between proteins and encoded genes, the nodes in PPI network we obtain are genes.

After collecting the above data, we use the union of genes in FANTOM[10], miRNAs in miRBase and genes in PPI as background genes to construct a network. The data from (1) to (8) sources are directly mapped to background genes according to the node name. If both nodes of an edge are in the background genes, the current edge is retained, otherwise, it is not retained. Finally, a network with 24215 nodes and 314748 edges without isolated points is obtained. We call this heterogeneous network IN (Interaction Network).

Supplementary Note S2. Cancer related expression data

We collect publicly available expression datasets from TCGA for 12 common cancers, including gene expression RNA-seq and miRNA mature strand expression RNA-seq [11-12]. According to the differences of data characteristics, DESeq2 [13] and Limma [14] are used to perform differential expression analysis for gene expression data and miRNA expression data, respectively. Finally, we get p-value and $\log_2 \mathrm{FC}$ for each gene or miRNA. For convenience, we denote $\log_2 \mathrm{FC}$ as $\log \mathrm{FC}$. FC refers to the fold change value. That is, the ratio of the expression value for a gene in the disease sample to the expression value in the normal sample is taken logarithmically to obtain FC for the gene. The log FC of a gene quantifies the degree to which the gene is affected by cancer. A gene whose $\log \mathrm{FC}$ is not 0 means that the expression of the gene is different between the disease samples and the normal samples, that is, the gene is differentially expressed. The greater the log FC of a gene deviates from 0, the higher the degree of differential expression of the gene. The number of samples related to each cancer is shown in Table S1.

Supplementary Table S1. The number of samples involved in gene expression data

Cancer Name	# Samples of gene expression RNAseq	# Samples of miRNA expression RNAseq
BLCA	427	429
BRCA	1215	832
COAD	328	261
ESCA	196	195
KIRC	606	311
KIRP	323	321
LIHC	217	420
LUAD	576	495
LUSC	553	380
READ	105	92
THCA	572	569
UCEC	190	430

Supplementary Note S3. The analysis of topological features

The network HIN is represented by $G = (V, E)$. $V = \{v_1, v_2, ..., v_n\}$ represents node set, which includes coding genes and non-coding genes. $E \subseteq V \times V$ represents interactions between genes. For a given cancer, the set of $|\log \mathrm{FC}|$ corresponding to genes are perturbed by cancer and is expressed as $F = \{f_1, f_2, ..., f_n\}$. We use the FC indicator ($f_g = |\log \mathrm{FC}|$) to quantify the level of differential expression and represent the degree to which node *g* is perturbed by cancer (f_g).

Any subgraph in *G* can be represented as $G_S = (V_S, E_S)$, where $V_S \in V$ and $E_S = \{(u,v) | (u,v) \in E, u \in V_S, v \in V_S\}$.

3.1 Connectivity significance test of cancer perturbed neighborhood

For the perturbed subgraph COModule or NeOModule of a given cancer, the statistical significance of its topological features is tested as follows.

To test whether a subgraph is a significantly connected or not in the network, we take $\mathrm{NeOModule}(\beta)$ as an example to illustrate. First, we randomly select 1,000 gene sets in HIN as counterparts. Each set has the same size and the proportion of gene types as $\mathrm{DE\_mRNAs}(\beta) \cup \mathrm{DE\_ncRNAs}$. Simultaneously, we quantified the connectivity of $\mathrm{NeOModule}(\beta)$ by measuring the size of the largest connected component (sLCC). Finally, the connectivity significance of is quantified by Z-score as:

$$\text{Z-score} = \frac{\mathrm{sLCC}_{\mathrm{true}} - \mu(\mathrm{sLCC}_{random})}{\sigma(\mathrm{sLCC}_{random})} \tag{1}$$

It should be noted the abscissa selection of Figure 2(a) is based on the following method. First, the set of perturbation degree values of all differentially expressed coding genes in a cancer is F_m. Then, we divide the values in set F_m into *t* values as thresholds ($t = 50$ in this paper). Next, denoting the obtained thresholds as $fs = \{fs_1, fs_2, ..., fs_{mt}\}$. We use equation (2) to define each threshold fs_i.

$$fs_i = \min(F_m) + \frac{\max(F_m) - \min(F_m)}{t} \times i, i = 1, 2, ..., t \tag{1}$$

Where $\max(F_m)$ and $\min(F_m)$ represent the maximum value and minimum value in

set F_m, respectively. Finally, $\forall fs_i \in fs$, we take fs_i as the perturbation threshold β for coding genes and obtain the connectivity significance of subgraph corresponding to each threshold as ordinate value in Figure 2(a).

3.2 Density of subgraph

The density of a subgraph G_S represents the denseness of edges in it, which is usually calculated by the equation (3).

$$Density = \frac{2|E_S|}{|V_S|(|V_S|-1)} \tag{2}$$

To test the statistical significance of the density, we compare the density of G_S with random subgraphs and quantify it with Z-score. First, the density of G_S is denoted as $OBS_{density}$. Next, we select the subgraph from network randomly and guarantee the size of random graph is the same as G_S. In addition, the number of connected component and the proportion of gene types in random subgraph are consistent with that in G_S.The density of each random subgraph is recorded as $REF_{density}$. The average $\mu(REF_{density})$ and standard deviation $\sigma(REF_{density})$ of $REF_{density}$ are used to calculate the statistical significance of subgraph density.

$$Z_{density} = \frac{OBS_{density} - \mu(REF_{density})}{\sigma(REF_{density})} \tag{3}$$

3.3 Conductance of subgraph

The conductance of subgraph is used to measure the interaction degree between internal nodes and external nodes of G_S, which is calculated by equation (5).

$$Conductance = \frac{|B_S|}{|B_S|+2|E_S|} \tag{4}$$

$B_S = \{(u,v) | (u,v) \in E, u \in V_S, v \in V \setminus V_S\}$ is the boundary set of G_S, V indicates nodes in the HIN network. The smaller the conductance value is, the less the subgraph is connected to its outside. That is, the tighter the subgraph structure is.

3.4 Spatial network association of subgraph

The spatial network association of G_S is used to measure the denseness of nodes in G, which is calculated by the shortest distance between nodes [15].

$$SpatialNA(l) = \frac{2}{(|V_S|)^2} \sum_i p_i \sum_j (p_j - \bar{p}) I(\mathcal{L}_G(i,j) < l) \tag{5}$$

If node i is in G_S, $p_i = 1$. Otherwise, $p_i = 0$. $\bar{p} = |V_S|/n$ and n is the number of nodes in G. If the shortest path length between i and j is less than l, then $I(\mathcal{L}_G(i,j) < l) = 1$. Otherwise, $I(\mathcal{L}_G(i,j) < l) = 0$. Here, a curve is formed by l from 2 to $l_{\max}$ and its corresponding value $SpatialNA(t)$. The area under the curve is denoted as $K(l)$. The larger $K(l)$ is, the subgraph G_S is more aggregated in the graph G. We set $l_{\max} = 5$ in our experiments according to the distance of nodes in HIN. The statistical significance quantification of conductance and spatial network association are similar to that of density. We denote their results as $Z_{conductance}$ and $Z_{spatialNA}$, respectively.

Supplementary Note S4. The functional profile of NeOModule

In order to investigate whether genes in NeOModule of a specific cancer are composed of genes with significant function, we analyze it with the help of a known data set. First of all, we collect four functional gene sets which mainly correlate with diseases and therapy of diseases. The detail information about four datasets is shown in Table S2. Then, we calculate the enrichment significance of genes in NeOModule on the four known datasets, and compare them with the enrichment results of other genes ($0 < |\log \mathrm{FC}| < \beta$) in this cancer that are differentially expressed but not within the threshold of perturbation. Finally, we quantify the enrichment results of different gene sets on the 4 functional gene datasets by p-values obtained from the hypergeometric distribution and excess overlap [16] between gene sets, respectively. When the p-value of the hypergeometric distribution is less than 0.05 and the excess overlap value is greater than 1, we consider genes in NeOModule are significantly enriched for functional genes.

Supplementary Table S2 The sources and numbers of functional gene dataset

gene sets	# genes	Source
GWAS	19110	http://www.ebi.ac.uk/gwas/
OMIM	16291	https://omim.org/
ClinVar [14]	5420	https://www.ncbi.nlm.nih.gov/clinvar/
Drug target [9]	2256	Network-based prediction of drug combinations

Supplementary Note S5. The subgraph centered on H19 in NeOModule

The process of obtaining H19-centered subgraph in NeOModule is as follows.

---

(1) **Input:**

A cancer neighborhood NeOModule

---

(2)**Process:**

Collect neighborhood of H19 in NeOModule as NE_{H19}

Record mRNAs in NE_{H19} as $NeighM$

While $|NeighM| \neq 0$

$\forall j \in NeighM$, collect neighborhood of $NeighM_j$

Record mRNAs in $\bigcup_{j=1}^{|NeighM|} NeighM_j$ as $NeighM$

$NE_{\mathrm{H19}} = NE_{\mathrm{H19}} \cup NeighM$

(3) **Output:**

The induced subgraph of NE_{H19} in NeOModule.

Supplementary Note S6. Module expansion

We record module of coding gene for a cancer collected from previous study as COModule. First, we obtain $\text{DE\_mRNAs}(1.5) \cup \text{DE\_ncRNAs}$ and the induced subgraph NeOModule corresponding to the set. Then, we get ncRNAs belong to neighborhoods of COModule in NeOModule and represent these ncRNAs as S_1.

$S_1 = \{v_2 | (v_1, v_2) \text{ in NeOModule}, v_1 \in COModule, v_2 \in \text{Neigh}(COModule), v_2 \in \text{ncRNAs}\}$

. The set of mRNAs in neighborhoods belong to ncRNAs is represented as S_2. Concretely, $S_2 = \{v_2 | (v_1, v_2), v_1 \in S_1, v_2 \in \text{Neigh}(S_1), v_2 \in \text{mRNAs}\}$. Finally, the gene set in module expanded by ncRNAs is $NeOModule = COModule \cup S_1 \cup S_2$.

Supplementary Note S7. The distance between cancer and drug

For a subgraph perturbed by cancer, we quantify the distance between the subgraph and each drug with proximity method. If the set of genes in the subgraph and the set of drug targets for a drug are represented as *S* and *T*. The distance beween both two sets can be calculated by equation (7).

$$d = \frac{1}{|T|} \sum_{t \in T} \min_{s \in S} d(s, t) \quad (7)$$

Then, by comparing the distances between the subgraph with random sets of drug targets with the same size, we obtain the statistic Z-score to measure how far the set *T* is from the subgraph *S*. Next, sorting drugs by Z-score and choose corresponding Z-score as threshold to predict the relationship between drug and cancer. Finally, the ROC curve and AUC score are calculated by obtaining FDA-approved drugs from repoDB as validation data.

Supplementary Figures

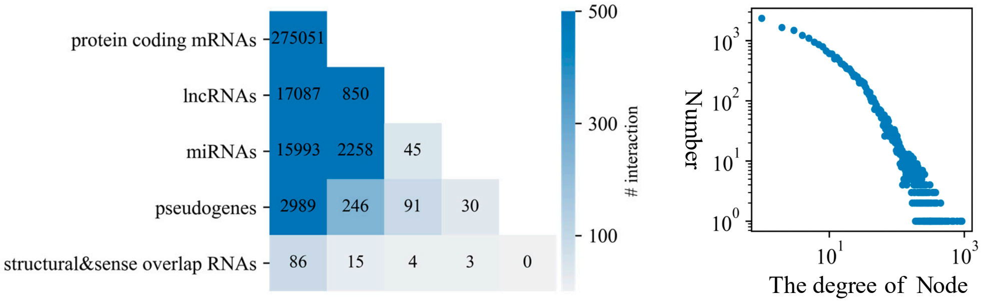

Figure S1. The details of interactions in the IN. The left figure shows the number of interactions among various genes in the network. Each number is the number of interactions between one gene type and the other gene type. For example, 15,993 is the number of interactions between protein-coding genes and miRNAs...The right figure shows degree distribution of nodes in the HIN. The degree of most nodes is small, which is consistent with the existing understanding of biological networks.

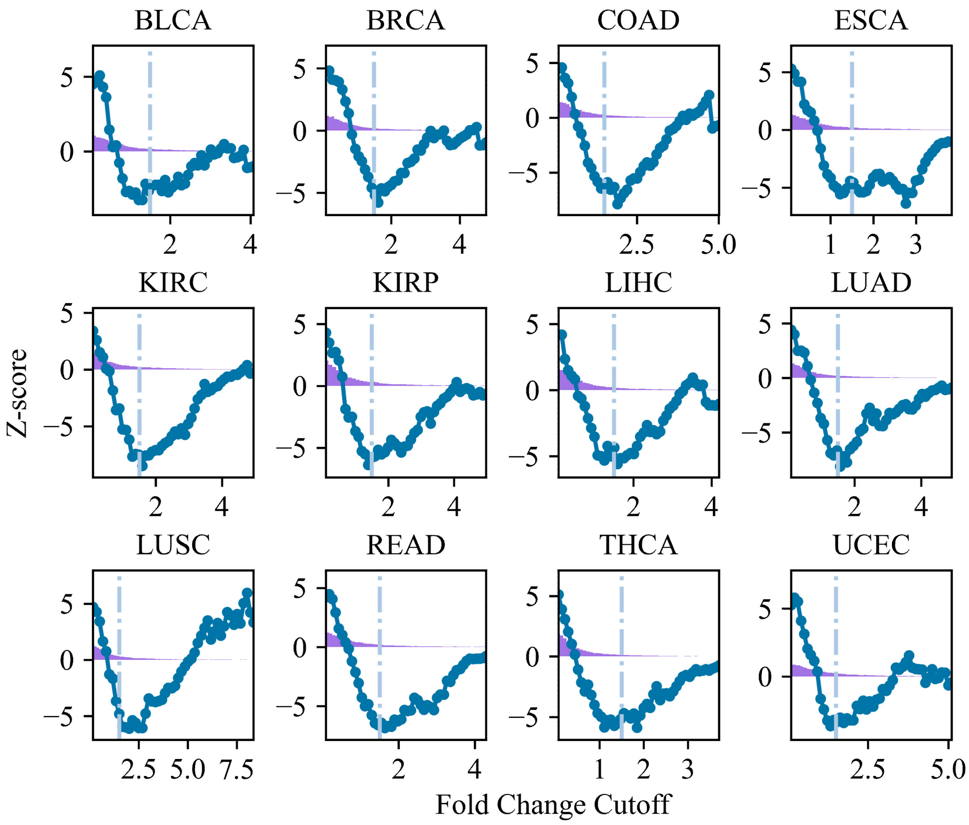

Figure S2. The connectivity curve of coding genes perturbed by each cancer. Each dot in this figure is the connectivity significance value of genes are perturbed when perturbation threshold is β. The abscissa of the light blue line is $\beta = 1.5$. The medium purple area in this figure represents the frequency distribution of the perturbed degree of all differentially expressed genes ($|\log FC| \neq 0$) in a specific cancer.

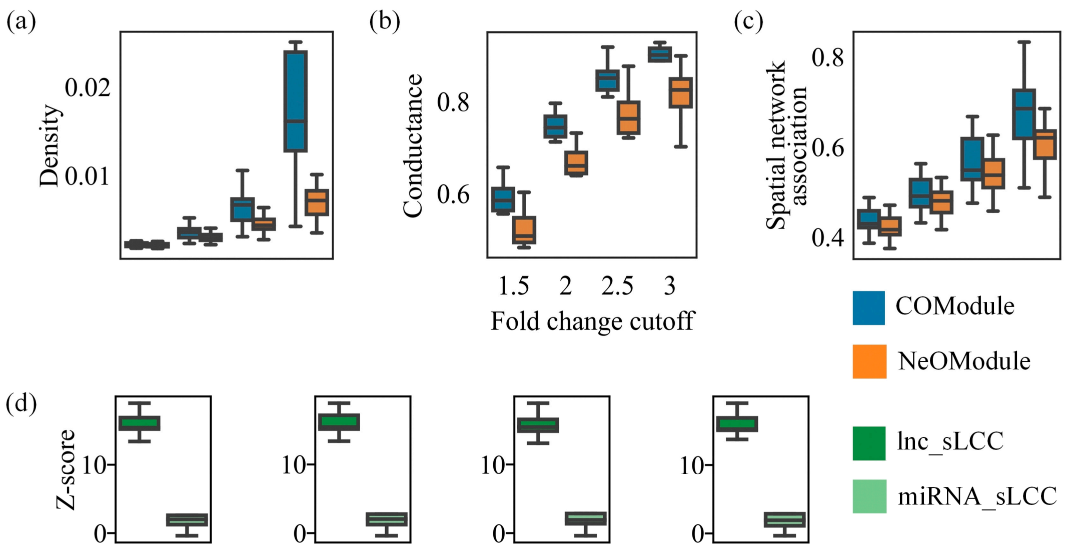

Figure S3. The topological features of COModule(β) and NeOModule(β). (a)-(c) The density, conductance and spatial network association of COModule and NeOModule for 12 cancers. (d)The connectivity significance Z-score of lncRNAs and miRNAs in HIN.

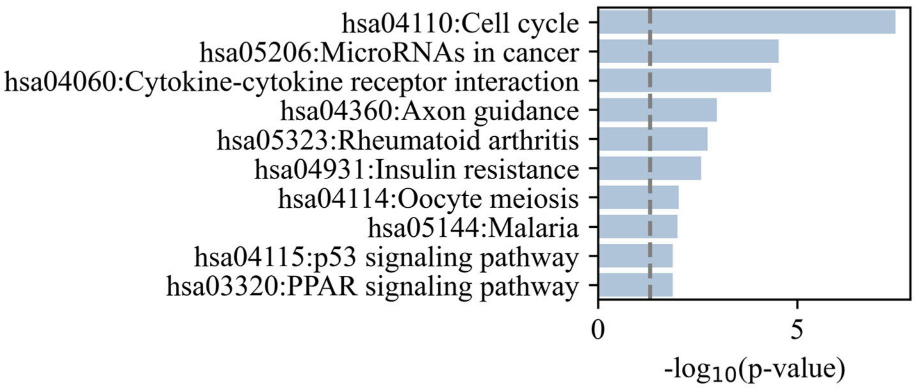

Figure S4. The top 10 pathways with the most least p-values enriched by NeOModule of BRCA.

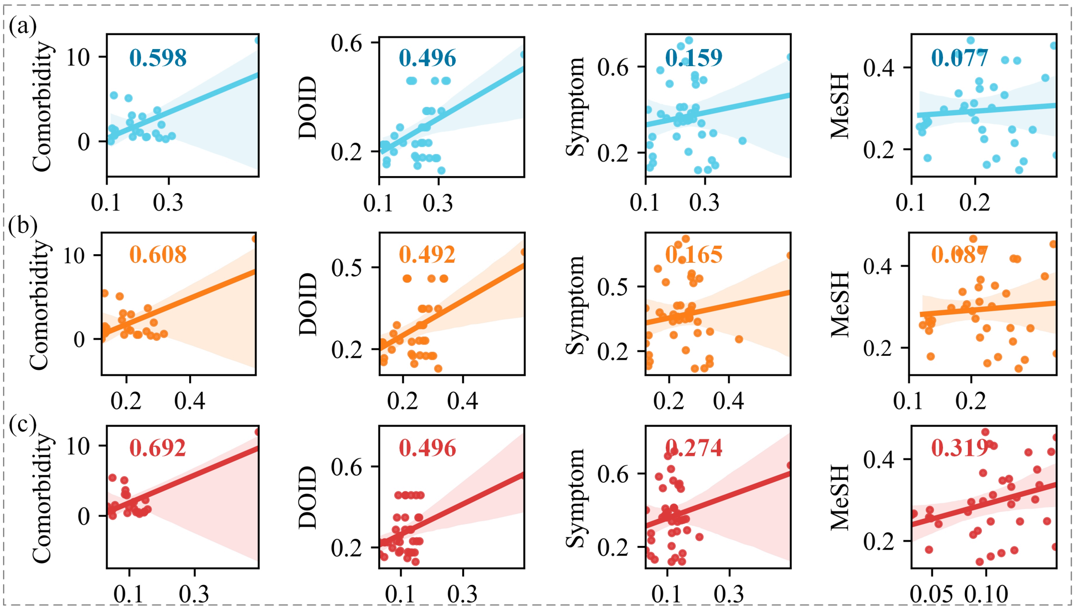

Fold change cutoff = 1.5

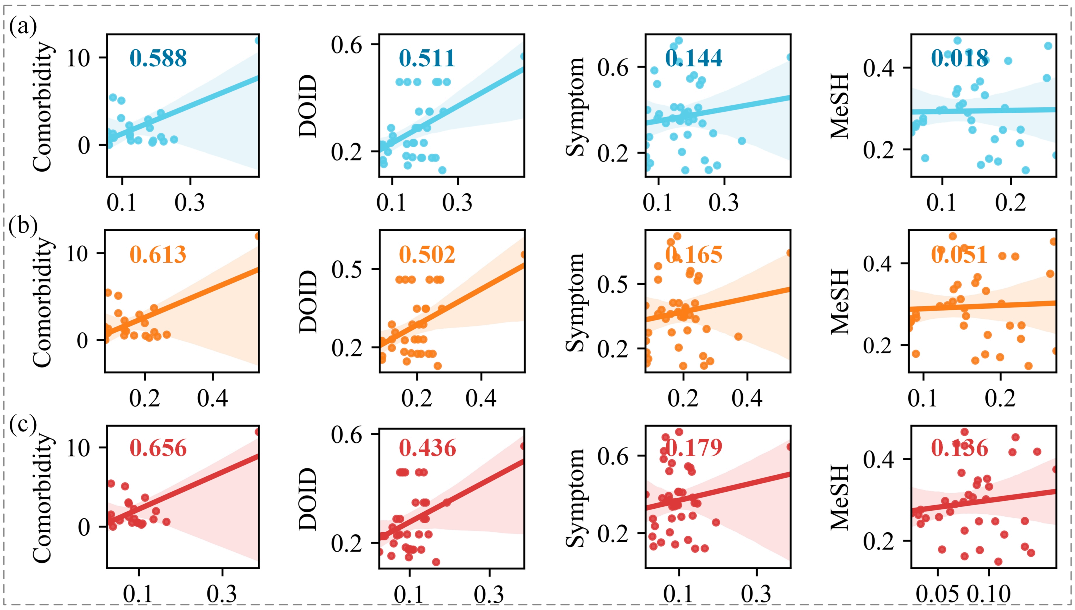

Fold change cutoff = 2

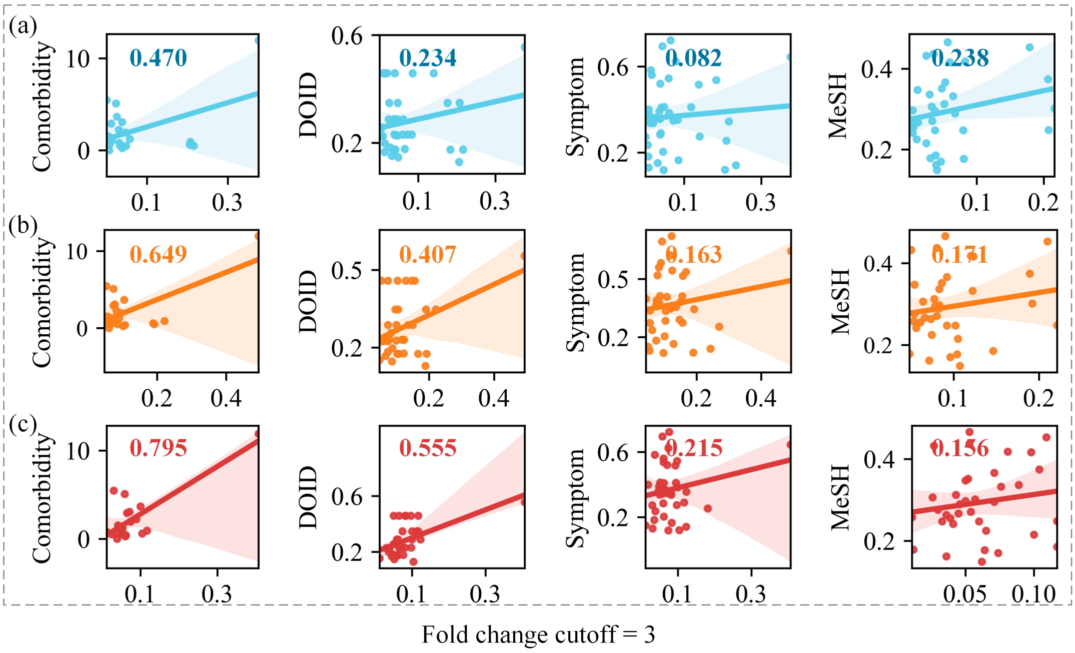

Figure S5. Associations between cancers characterized by NeOModule, COModule and Iso_mRNAs of 12 cancers and the correlation between similarities calculated by our study and similarities from existing studies under different perturbation thresholds.

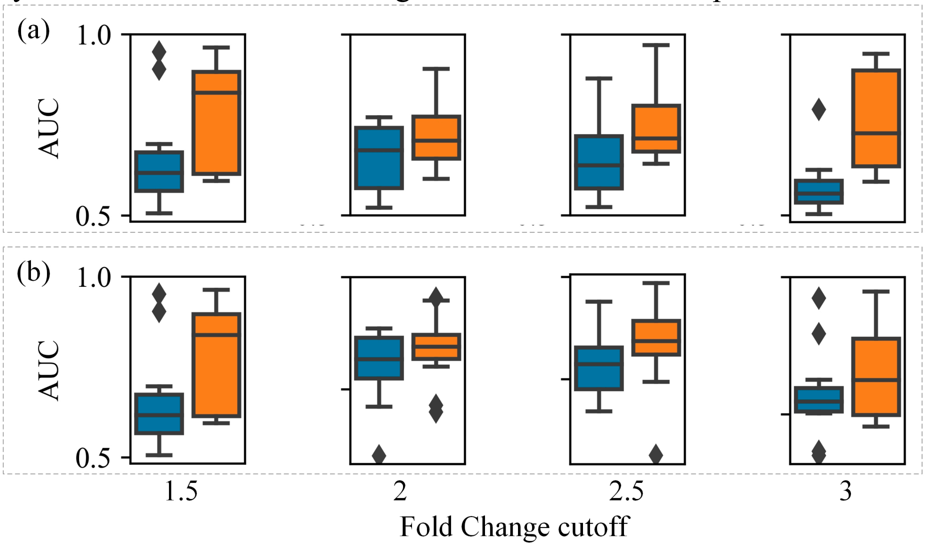

Figure S6. Performance of COModule and NeOModule in drug prediction for 12 cancers under different perturbation thresholds. (a) Boxplots for cancer outcomes of drug prediction AUC greater than 0.5 with both COModule and NeOModule. The AUC of COModule and NeOModule in drug prediction of 12 (100%), 10 (83.33%), 8 (66.67%), 8 (66.67%) cancers are greater than 0.5 respectively when $\beta \in \{1.5, 2, 2.5, 3\}$. (b) AUC of COModule and NeOModule in drug prediction for 12 cancers.

Supplementary References

1. Lin Y, Liu T, Cui T, Wang Z, Zhang Y, Tan P, Huang Y, Yu J, Wang D. 2020 RNAInter in 2020: RNA interactome repository with increased coverage and annotation. *Nucleic Acids Res.* **48**, D189–D197. (doi:10.1093/nar/gkz804)
2. Xiao F, Zuo Z, Cai G, Kang S, Gao X, Li T. 2009 miRecords: An integrated resource for microRNA-target interactions. *Nucleic Acids Res.* **37**, 105–110. (doi:10.1093/nar/gkn851)
3. Chen G, Wang Z, Wang D, Qiu C, Liu M, Chen X, Zhang Q, Yan G, Cui Q. 2013 LncRNADisease: A database for long-non-coding RNA-associated diseases. *Nucleic Acids Res.* **41**, 983–986. (doi:10.1093/nar/gks1099)
4. Huang HY *et al.* 2020 MiRTarBase 2020: Updates to the experimentally validated microRNA-target interaction database. *Nucleic Acids Res.* **48**, D148–D154. (doi:10.1093/nar/gkz896)
5. Chatr-Aryamontri A *et al.* 2015 The BioGRID interaction database: 2015 update. *Nucleic Acids Res.* **43**, D470–D478. (doi:10.1093/nar/gku1204)
6. Li X *et al.* 2019 OncoBase: A platform for decoding regulatory somatic mutations in human cancers. *Nucleic Acids Res.* **47**, D1044–D1055. (doi:10.1093/nar/gky1139)
7. Zhu Y *et al.* 2016 Constructing 3D interaction maps from 1D epigenomes. *Nat. Commun.* **7**, 1–11. (doi:10.1038/ncomms10812)
8. Wang P *et al.* 2019 LncACTdb 2.0: an updated database of experimentally supported ceRNA interactions curated from low- and high-throughput experiments. *Nucleic Acids Res.* **47**, D121–D127. (doi:10.1093/nar/gky1144)
9. Cheng F, Kovács IA, Barabási AL. 2019 Network-based prediction of drug combinations. *Nat. Commun.* **10**, 1197. (doi:10.1038/s41467-019-09186-x)
10. Hon C C , Ramilowski J A , Harshbarger J ,et al.An atlas of human long non-coding RNAs with accurate 5' ends[J].Nature, 2017, 543(7644):199-204.(doi:10.1038/nature21374)
11. Fu G, Wang J, Domeniconi C, Yu G. 2018 Matrix factorization-based data fusion for the prediction of lncRNA-disease associations. *Bioinformatics* **34**, 1529–1537. (doi:10.1093/bioinformatics/btx794)
12. Li W, Deng G, Zhang J, Hu E, He Y, Lv J, Sun X, Wang K, Chen L. 2019 Identification of breast cancer risk modules via an integrated strategy. *Aging (Albany. NY).* **11**, 12131–12146. (doi:10.18632/aging.102546)

13 Love M I , Huber W , Anders S .Moderated estimation of fold change and dispersion for RNA-seq data with DESeq2[J].Genome Biology, 2014, 15(12):550.(doi:10.1186/s13059-014-0550-8)

14 Ritchie M E , Belinda P , Di W ,et al.limma powers differential expression analyses for RNA-sequencing and microarray studies[J].Nucleic acids research, 2015, 43(7):e47.(doi:10.1093/nar/gkv007)

15. Agrawal M, Zitnik M, Leskovec J. 2018 Large-scale analysis of disease pathways in the human interactome. *Pacific Symp. Biocomput.* **0**, 111–122. (doi:10.1142/9789813235533_0011)
16. Kim SS *et al.* 2019 Genes with High Network Connectivity Are Enriched for Disease Heritability. *Am. J. Hum. Genet.* **104**, 896–913. (doi:10.1016/j.ajhg.2019.03.020)
